# Unusual localization of a primary pleomorphic malignant fibrous histiocytoma on the mitral valve: a case report and review of the literature

**DOI:** 10.1186/s13256-015-0726-1

**Published:** 2015-10-31

**Authors:** Edvin Prifti, Fadil Ademaj, Majlinda Ikonomi, Aurel Demiraj

**Affiliations:** 1 Division of Cardiac Surgery, University Hospital Center of Tirana, Tirana, Albania; 2 Division of Cardiology, Regional Hospital of Gjakovo, Gjakovo, Kosovo; 3 Division of Heart Disease, Gjakovo Hospital, Rr. Prizren, Gjakove, Kosove

**Keywords:** Left atrium, Mitral valve, Pleomorphic malignant fibrous histiocytoma

## Abstract

**Introduction:**

It has been reported that cardiac malignant fibrous histiocytomas occur more frequently in the left side of the heart, especially in the left atrium, but rarely invade the mitral valve. We present a case with a giant malignant fibrous histiocytoma with an unusual localization involving almost the entire left atrium, mitral valve, and left superior pulmonary vein.

**Case presentation:**

We describe the case of a 54-year-old woman from Kosovo admitted to our emergency department with dyspnea. A transthoracic echocardiography demonstrated a giant mass localized on her left atrium. Our patient underwent emergent total surgical removal of the mass. The mass extended between her left superior pulmonary vein, and extended to her left atrium and the posterior mitral valve leaflet. We formulated a surgical plan for total separation of the mass from the endocardium. Total removal was performed and her left side pulmonary veins were entirely freed from the mass. We then performed a mitral valve replacement. The differential diagnosis included other masses of the left atrium, including thrombi, vegetations, and cardiac tumors. Postsurgical histopathologic results showed a pleomorphic malignant fibrous histiocytoma. Six monthly follow-up cardiac and abdominal sonographic examinations revealed no tumor recidivism.

**Conclusion:**

We reviewed 90 cases with malignant fibrous histiocytoma reported in the literature. Our case was especially unusual because of the primary location in the mitral valve, the pleomorphic variant, and the dimensions and extension. Complete surgical resection is mandatory to ameliorate symptoms and to obtain histologic information.

## Introduction

The first reported case of a cardiac malignant fibrous histiocytoma (MFH) was published by O’Brien *et al*. [1] in 1964 and the first case undergoing surgical removal in 1978 [2]. In 2001, Okamoto *et al*. [3] analyzed 46 cases of reported MFH. Since then, 44 additional cases of MFH have been reported, resulting in a total of 90 cases of MFH. Cardiac MFH occur more frequently in the left side of the heart, especially in the left atrium [4–6], but rarely invade the mitral valve. The pleomorphic variant of MFH is rarely found and comprises a high cellularity with bizarre tumor cells with marked atypia and high mitotic index. Here, we report the case of a patient with a pleomorphic MFH invading her mitral valve that underwent successful surgical removal. To the best of our knowledge, this is the first reported case of a pleomorphic variant of such dimensions and tumor extension involving the mitral valve.

## Case presentation

A 54-year-old woman from Kosovo was admitted to our hospital with dyspnea. On clinical examination, our patient’s blood pressure was 100/60 mmHg, and her pulse rate was 130 beats per minute with a regular rate and rhythm. Cardiac auscultation revealed a diastolic murmur. End-inspiratory crackles suggested pulmonary edema. Two-dimensional transthoracic echocardiography revealed a giant mass originating from her posterior mitral valve leaflet, occupying almost her entire left atrial cavity. Cerebral, thoracic, and abdominal computed tomography was also performed, showing no evidence of additional tumors.

**Fig. 1:**
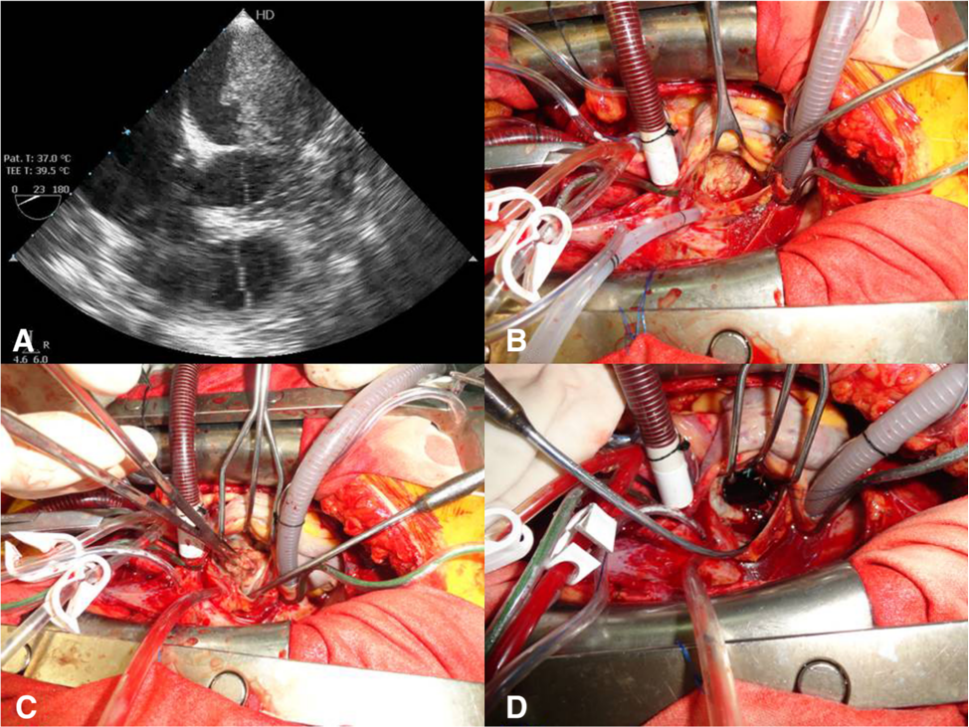
**a** Intraoperative transesophageal echocardiography. **b** Yellowish-white mass in the left atrium. **c** Removal of the mass from the endocardium. **d** Mitral valve replacement with a mechanical prosthesis

Our patient underwent emergency surgical removal of the cardiac tumor. Intraoperative transesophageal echocardiography was performed, which confirmed the presence of the tumor (Fig. 1a). Our patient underwent bicaval cannulation. Her aorta was clamped and anterograde cardioplegia was administered. Then her left atrium was opened at the interatrial groove. A giant yellowish-white tumoral mass was identified, occupying almost her entire left atrial cavity (Fig. 1b). The mass had invaded her left superior pulmonary vein and extended into the posterior aspect of her left atrium. The tumor invaded the posterior leaflet of her mitral valve. The tumor mass was carefully detached from the endocardium (Fig. 1c) and then entirely removed, including the posterior leaflet of her mitral valve, which was replaced with a 29-mm St Jude mechanical prosthesis (Fig. 1d).

**Fig. 2:**
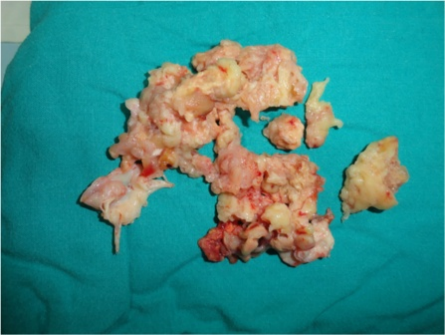
The excised macroscopic tumoral fragments

**Fig. 3:**
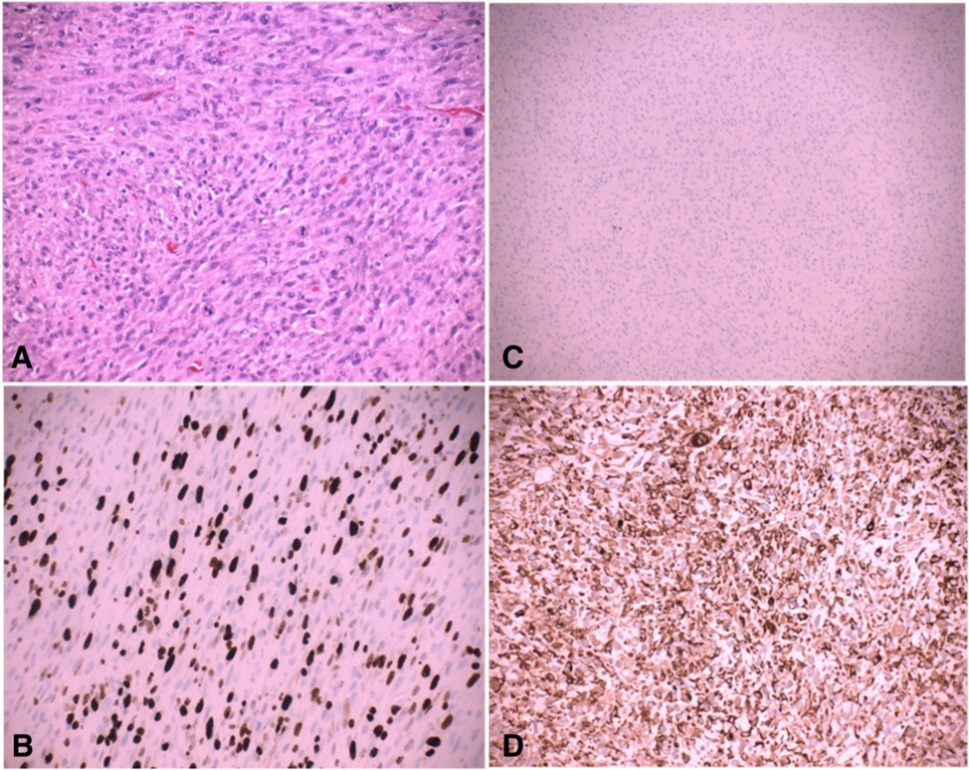
**a** Histological examination revealed a high grade sarcoma with a fusicellular proliferation organized in a partial storiform pattern, with irregular fascicles, high cellularity, and pleomorphic and bizarre tumor cells with marked atypia and a high mitotic index (hematoxylin and eosin ×*20*). **b** Staining was 25 % positive for Ki-67 in the tumor (Ki-67 ×20). **c** The resected sample was negative for muscle markers (smooth muscle actin *×*20) and melanocytic markers CD45 and S10. **d** The immunohistochemical examinations were positive for CD68, vimentin, and alpha-1-antitrypsin (CD68 ×20)

Macroscopically, the excised lesion was composed of multiple irregular soft tissue fragments (Fig. 2). After the surgical excision, the mass was fixed in formalin, paraffin embedded, sectioned at 3-μm thick, and stained conventionally with hematoxylin and eosin. Examination of the histology revealed a high grade sarcoma composed of a fusicellular proliferation in a partial storiform pattern, with irregular fascicles, high cellularity, and pleomorphic and bizarre tumor cells with marked atypia and a high mitotic index (Fig. 3a). There were also large areas of necrosis. Immunohistochemical examination results were 25 % positive for Ki-67 in the tumor (Fig. 3b); negative for the muscle markers and the melanocytic markers CD45 and S100 (Fig. 3c); and positive for CD68, vimentin, and alpha-1-antitrypsin (Fig. 3d). A diagnosis of pleomorphic MFH was made.

Our patient’s postoperative course was uneventful. Six weeks after surgery, she started a six-course chemotherapy regimen of 1.5 mg/m^2^ of ifosfamide on days 1 to 3 and 80 mg/m^2^ of epidoxorubicin on day 1. The treatment was well tolerated with no unacceptable toxicities. Our patient was still alive with no signs of metastasis six months later.

## Discussion

Primary MFH is the second most common primary cardiac sarcoma, with an estimated incidence of ∼ 1.7 % of cases. It usually affects people of ages 14 to 77 years [3]. After a careful review of the literature, we found reports of a total of 90 cases of primary MFH (Table 1). Primary MFH is common in the left heart, and it frequently manifests as an embolism. Almost 65.5 % of the reported patients presented with a primary MFH in the left atrium. The tumor can also localize in different portions of the left atrium, such as the pulmonary veins [4], septum, and posterior wall. However, the MFH invaded the mitral valve in only eight of the 90 cases [7–13], including our own. In our case, the tumor occupied almost her entire left atrial cavity. Our report seems to be the first reported case with such large tumor dimensions and extension involving the pulmonary vein, left atrium, and mitral valve. The non-septal origin of the mass strongly supports the suspicion of sarcoma. Multiple attachment sites and infiltration of the mitral valve and the atrial and ventricular walls are also indicative of malignancy.

**Table 1:** Cases reported in the literature with primary cardiac malignant fibrous histiocytoma

Variables	
Total	90
Female	60 (67 %)
Age (years)	14–80
Localization	
Left atrium	49
Pericardium	5
Pulmonary veins	2
Mitral valve	8
Right ventricle	8
Right atrium	9
Left ventricle	5
Pulmonary artery	3
Inferior vena cava	1
Symptoms	
Dyspnea	63 (70 %)
Palpitations	40 (45 %)
Pleomorphic	12 (13 %)

Clinical manifestations of primary MFH are dependent on their size and location. With small tumors, no clinical manifestations are evident. As the tumor grows, the most frequent symptoms are shortness of breath, palpitation, or chest discomfort. The MFH in our case is, to the best of our knowledge, the largest reported.

Histologically, MFH is a variously shaped and multilobulated mass, sessile or pedunculated. It is a tumor of the fibroblasts, with giant cells and atypical nuclear and cytoplasmic findings. The term MFH has now become synonymous of undifferentiated high-grade pleomorphic sarcoma, because the fibrohistiocytic differentiation is not characteristic of a specific tumor type. Sarcomas of myoblastic or fibroblastic origin, including leiomyosarcoma, fibrosarcoma, myxoid fibrosarcoma, and pleomorphic sarcoma (MFH), occur most often as endocardium-based pathologies. The differential diagnosis of cardiac sarcoma in these circumstances is cardiac myxoma, especially in the case of a left atrial intimal-type sarcoma. MFH is a pleomorphic sarcoma composed of fibroblasts, myofibroblasts, and histiocyte-like cells. The pleomorphic variant of the MFH is rarely reported and only had mitral valve involvement in two of the 90 cases we found in the literature [8, 9].

The treatment plan for MFH depends on the tumor status, size, histology, location, and metastatic spread. Complete surgical resection is mandatory to ameliorate symptoms and to obtain histologic information. In our case, once we had started surgery, we suspected that we had a malignant tumor. However, owing to the fact that the tumor had invaded her posterior mitral valve leaflet, causing severe mitral valve obstruction, we had to remove her mitral valve as this was the only measure to complete total tumoral mass removal and relieve the mitral valve stenosis.

Although the prognosis of MFH is poor and the possibility of local recurrence and metastasis is high, patients benefit from surgery. Multiple studies have reported a median survival of six months for right-sided tumors, whereas left heart tumors seem to have a better prognosis [14]. The role of chemotherapy and radiotherapy in the treatment of primary cardiac sarcomas has not proven to be beneficial, and complete surgical excision is the only mode of therapy that has been shown to prolong survival.

## Conclusion

Our case was especially unusual because of the primary location of an MFH in the mitral valve, the pleomorphic variant, and the dimensions and extension. Complete surgical resection, including mitral valve replacement, is mandatory to improve symptoms and to obtain histologic information.

## Consent

Written informed consent was obtained from the patient for publication of this case report and any accompanying images. A copy of the written consent is available for review by the Editor-in-Chief of this journal.
